# PpASCL, the *Physcomitrella patens* Anther-Specific Chalcone Synthase-Like Enzyme Implicated in Sporopollenin Biosynthesis, Is Needed for Integrity of the Moss Spore Wall and Spore Viability

**DOI:** 10.1371/journal.pone.0146817

**Published:** 2016-01-11

**Authors:** Rhys M. Daku, Fazle Rabbi, Josef Buttigieg, Ian M. Coulson, Derrick Horne, Garnet Martens, Neil W. Ashton, Dae-Yeon Suh

**Affiliations:** 1 Department of Chemistry and Biochemistry, University of Regina, Regina, Saskatchewan, Canada; 2 Department of Biology, University of Regina, Regina, Saskatchewan, Canada; 3 Department of Geology, University of Regina, Regina, Saskatchewan, Canada; 4 BioImaging Facility, University of British Colombia, Vancouver, British Columbia, Canada; University of Birmingham, UNITED KINGDOM

## Abstract

Sporopollenin is the main constituent of the exine layer of spore and pollen walls. The anther-specific chalcone synthase-like (ASCL) enzyme of *Physcomitrella patens*, PpASCL, has previously been implicated in the biosynthesis of sporopollenin, the main constituent of exine and perine, the two outermost layers of the moss spore cell wall. We made targeted knockouts of the corresponding gene, *PpASCL*, and phenotypically characterized *ascl* sporophytes and spores at different developmental stages. *Ascl* plants developed normally until late in sporophytic development, when the spores produced were structurally aberrant and inviable. The development of the *ascl* spore cell wall appeared to be arrested early in microspore development, resulting in small, collapsed spores with altered surface morphology. The typical stratification of the spore cell wall was absent with only an abnormal perine recognisable above an amorphous layer possibly representing remnants of compromised intine and/or exine. Equivalent resistance of the spore walls of *ascl* mutants and the control strain to acetolysis suggests the presence of chemically inert, defective sporopollenin in the mutants. Anatomical abnormalities of late-stage *ascl* sporophytes include a persistent large columella and an air space incompletely filled with spores. Our results indicate that the evolutionarily conserved *PpASCL* gene is needed for proper construction of the spore wall and for normal maturation and viability of moss spores.

## Introduction

The transition of plants from aquatic to terrestrial environments about 500 million years ago is one of the most important events in the evolution of life on earth. On land, plants encountered new challenges, which included surviving exposure to higher fluxes of damaging UV irradiation and desiccation. Plants successfully adapted to these new environments by, among other things, producing novel secondary metabolites: phenylpropanoids for protection from UV rays and extracellular matrices of sporopollenin and cutin to counter desiccation [1, 2]. Discovering additional information about evolution of the biosynthetic pathways of these protective compounds will contribute significantly to our comprehension of the colonization of land by early plants.

**Fig 1:**
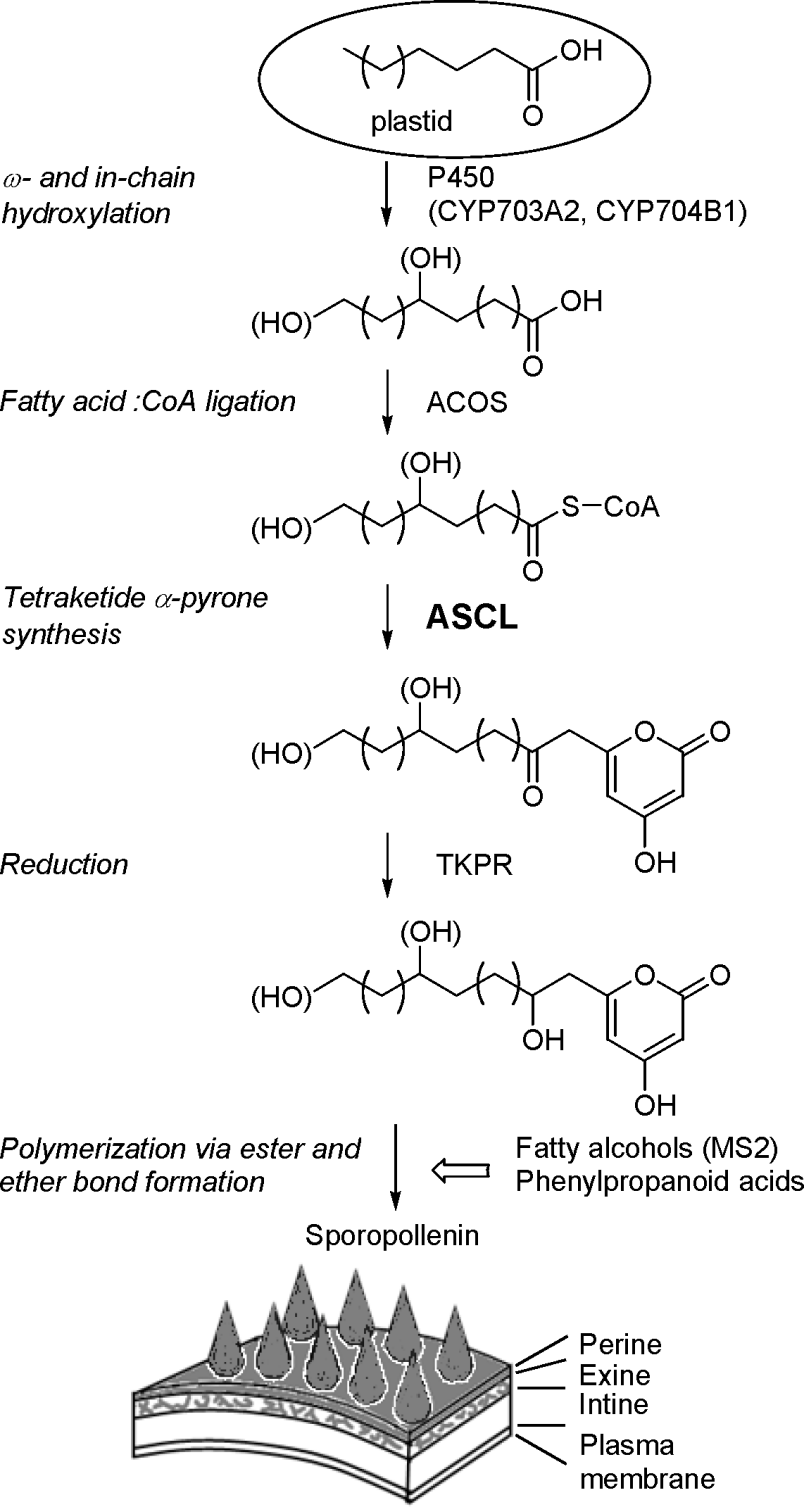
Reaction sequence for the biosynthesis of hydroxylated alkylpyrones as sporopollenin building blocks. Medium- to long-chain fatty acids are produced in plastids and then translocated out to be used for the consecutive action of enzymes in sporopollenin biosynthesis. This proposed pathway produces sporopollenin building blocks that are polymerized along with fatty alcohols and phenylpropanoid acids on the surface of the spore or pollen wall by the formation of ester and ether linkages. Enzymes are listed to the right of the arrows with their corresponding reactions on the left. ACOS, acyl-CoA synthetase; ASCL, anther-specific chalcone synthase-like enzyme; MS2, Male Sterility 2; TKPR, tetraketide α-pyrone reductase.

Sporopollenin is the main polymeric component of the outer exine layer of spore and pollen walls, and consists of medium- to long-chain fatty acids and oxygenated aromatic compounds [3]. These constituents are coupled via extensive ester and ether linkages, resulting in a robust polymer that enables spore and pollen grains to tolerate physical abrasion, desiccation and UV-B irradiation [4, 5]. Recently, several genes have been shown to be involved in the biosynthesis of sporopollenin (Fig 1) [6]. The *MALE STERILITY2* (*MS2*) gene encodes a fatty acyl reductase that reduces very long chain fatty acyl-CoA or acyl-acyl carrier protein to fatty alcohol [7, 8]. The cytochrome P450 genes, *CYP703A2* and *CYP704B1*, encode fatty acid hydroxylases that catalyze in-chain and ω-hydroxylation respectively of mid- to long-chain fatty acids [9, 10]. Fatty alcohols and hydroxyfatty acids produced by these gene products, along with phenylpropanoids (e.g. *p*-coumaric acid and ferulic acid) [11], may serve as building blocks and provide oxygen atoms for ester and ether linkages in sporopollenin. In addition, the hydroxylated alkylpyrones generated by sequential actions of acyl-CoA synthetase, anther-specific chalcone synthase-like enzyme (ASCL) and tetraketide α-pyrone reductase are either direct sporopollenin monomers or incorporated into sporopollenin after further modifications [12, 13, 14]. ASCL is a type III polyketide synthase (PKS), and produces *in vitro* hydroxyalkyl α-pyrones by condensing hydroxyfatty acyl-CoA esters with malonyl-CoA molecules [14]. In Arabidopsis, *PKSA* and *PKSB*, encoding two paralogous ASCLs [15], are specifically expressed in anther tapetal cells during microspore development. A double *pksa pksb* knockout mutant was male sterile and produced defective pollen grains with no apparent exine, providing evidence for the involvement of ASCL in sporopollenin biosynthesis [14].

Bryophytes (liverworts, mosses, and hornworts) are the simplest and earliest-diverging lineages of land plants. The phylogeny of land plants supports the hypothesis that early land plants were bryophyte-like and thus bryophytes offer unique windows into the early evolution of land plants [16]. The moss *Physcomitrella patens* has emerged as a model bryophyte [17]. *Physcomitrella* is unique among plants in that targeted gene knockouts can be efficiently produced by homologous recombination [18]. Loss-of-function phenotypes can be readily screened at all stages of development after mutated protoplasts derived from haploid protonemal cells are regenerated. These characteristics render *Physcomitrella* an excellent system for studying plant physiology and early land plant evolution with reverse genetics approaches. Consequently, the genome of *Physcomitrella* has been sequenced, and large-scale gene expression profiling data are available from different tissues of the plant [19, 20].

The presence of sporopollenin in the outer cell wall layers of pollen and spores of land plants indicates that the development of this resistant biopolymer occurred early in land plant evolution. The genome of *Physcomitrella* contains putative orthologues of the sporopollenin biosynthesis genes, suggesting that the biosynthetic pathway of sporopollenin may be conserved in land plant lineages [21, 22]. *PpASCL* is the putative *Physcomitrella* orthologue of Arabidopsis *PKSA* and *PKSB*. EST abundance data and gene expression analysis by microarrays suggested that *PpASCL* is specifically expressed in the green sporophyte stage of development. Similar to PKSA and PKSB, recombinant PpASCL preferred hydroxylated fatty acyl-CoA esters as the starter substrate and produced hydroxyalkyl α-pyrones *in vitro* [21]. These results suggested that PpASCL may also play a role in the production of sporopollenin in *Physcomitrella*. An *ascl* loss-of-function mutant is then expected to have altered spore wall formation, similar to the pollen phenotype of *pksa* and *pksb* single and double mutants of Arabidopsis [14]. In this study, targeted *PpASCL* knockout transformants were made to elucidate the *in planta* function of PpASCL. Phenotypic characterization of the *Physcomitrella ascl* mutants supports a role for *PpASCL* in spore wall development, in agreement with its proposed participation in the evolutionarily conserved sporopollenin biosynthetic pathway.

## Materials and Methods

### Plant material, media and culture conditions

The *Physcomitrella* lines used in this study were characterized by an isogenic genetic background. Transgenic strains were derived from the *pabB4* [23] control strain of *P*. *patens* (Hedw.) Bruch, Schimp & W. Gümbel. When grown on medium containing adequate (1.8 and 18 μM for gametophytes and sporophytes respectively) *p*-aminobenzoic acid (paba), *pabB4* is phenotypically indistinguishable from the original Gransden wild type strain from which it was derived. It produces abundant sporophytes containing viable spores with normal spore coat ornamentation and completes its life-cycle in 2–3 months [24].

Plants were grown axenically on solid ABC medium [25] supplemented with paba (1.8 μM) at 22–25°C. Tissues for DNA and protoplast isolation were grown on medium including diammonium (+)-tartrate (5 mM) and overlaid with cellophane discs [26]. Filter-sterilized G418 (50 μg ml^-1^) was added as necessary to autoclaved medium. Continuous light culture conditions, described previously [27], were utilised with a single modification: Petri plates were covered with one layer of resin filter [Roscolux No. 114 (Hamburg frost), MacPhon Industries, Calgary, AB, Canada].

### Preparation of linear *PpASCL* knockout construct

**Fig 2:**
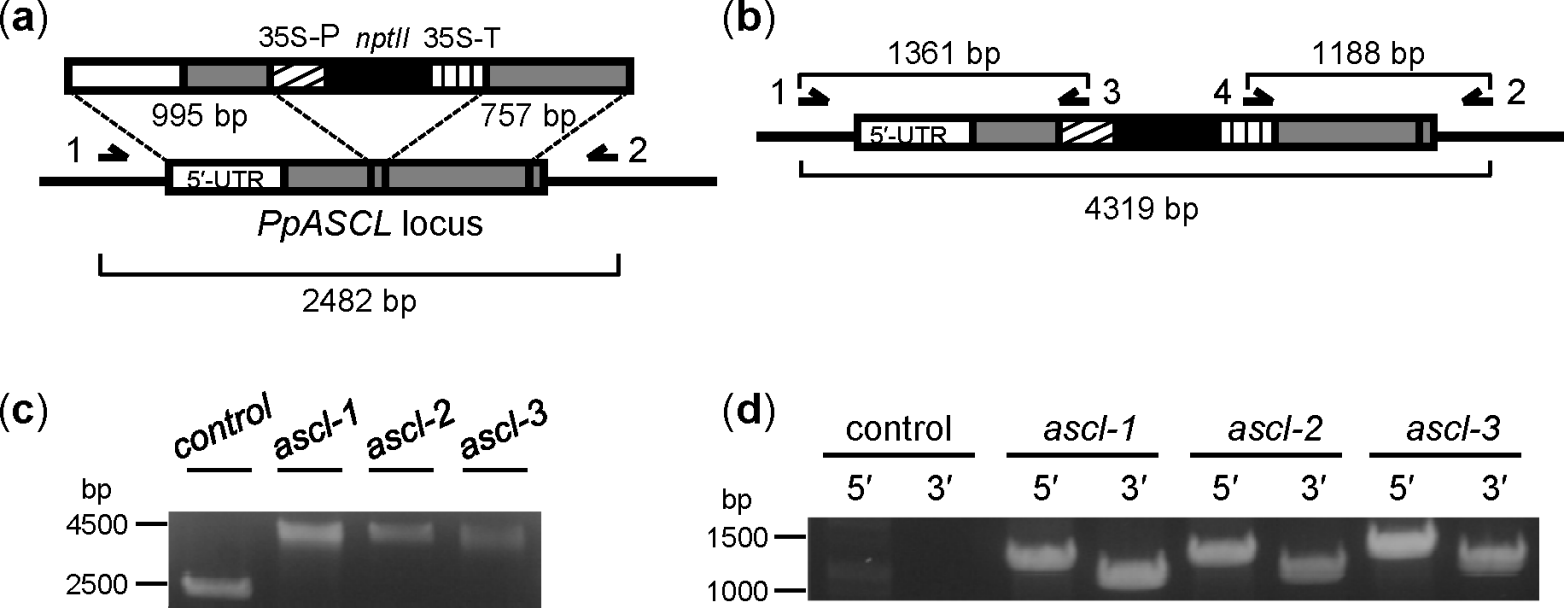
Strategy for targeted knockout of *PpASCL* and genotyping of the resulting stable transformants by PCR. (a) Schematic diagram of insertion of the linear knockout construct into the *PpASCL* locus via double homologous recombination. 35S-P, CaMV 35S promoter; *nptII*, neomycin phosphotransferase II gene; 35S-T, CaMV 35S transcription termination signal. (b) Schematic diagram of recombined gene locus after successful insertion. Expected PCR product sizes based on sequence information are shown. Single-headed arrows denote the locations of primers specific to *PpASCL* (Primers 1 and 2, which bind to genomic DNA sequences located outside the locus-specific regions used for homologous recombination) or to the *nptII* resistance cassette (Primers 3 and 4) used in the PCR analyses. Primer 1, ASCL-gDNA-F; 2, ASCL-gDNA-R; 3, pTN182-5ʹ-R; 4, pTN182-3ʹ-F. Primer sequences are provided in S1 Table. (c) PCR products using locus-specific primers 1 and 2 with DNA from untransformed control and each of three stable putative *PpASCL* knockout lines: *ascl-1*, *-2* and *-3*. (d) PCR products, indicative of 5′ and 3′ recombination between the knockout vector and homologous DNA in the *PpASCL* locus, using primers 1 plus 3 (5ʹ recombination) and primers 2 plus 4 (3ʹ recombination). Amplified DNA products were resolved electrophoretically on 1.2% agarose gels and visualized by ethidium bromide fluorescence.

The knockout construct was derived using pTN182 [28]. This plasmid has a resistance cassette with a neomycin phosphotransferase II gene (*nptII*), which confers resistance to a range of aminoglycoside antibiotics including G418. 5′ and 3′ regions of *PpASCL* were PCR-amplified from *Physcomitrella* genomic DNA (gDNA) using the 5ʹF- and 5ʹR-ASCL-ClaI primer pair and 3ʹF- and 3ʹR-ASCL-NdeI primer pair respectively. The 995 bp 5' locus-specific DNA fragment comprised 549 bp of 5' untranslated region and 446 bp of protein coding sequence while the 757 bp 3′ locus-specific DNA fragment entirely comprised of coding DNA (Fig 2A). The primers used in this study are listed in S1 Table. The two PCR products were appropriately restricted and consecutively ligated into *Cla*I- and *Nde*I-restricted pTN182 plasmid to produce the knockout vector, pTN182-PpASCL-KO. Restriction and PCR analyses, as well as sequencing, were used to confirm the desired insertion and orientation of the two locus-specific regions of *PpASCL*.

Linear knockout DNA was PCR-amplified from pTN182-PpASCL-KO using 5′F-ASCL-ClaI and 3′R-ASCL-NdeI. The amplified construct was gel purified, ethanol precipitated, resuspended in sterile water and then used directly for protoplast transformation.

### Transformation and selection of transgenic plants

Direct transfer of linear knockout constructs (approx. 20 μg in each case) into *pabB4* protoplasts and selection of stable transformants were performed as previously described [29] except for the nature of transforming DNA used. Stable transformants were defined as those that retained their transformed phenotype (resistance to G418) after two successive rounds of culturing for two weeks each on non-selective medium before re-culturing in the presence of the selective agent [24].

### Molecular analysis of transgenic plants: PCR genotyping, Southern blot and RT-PCR

gDNA was extracted from 7–14 day old protonemal tissue using a DNeasy Plant kit (Qiagen Inc., Toronto, ON, Canada). Insertion of knockout DNA into *PpASCL* in stable transformants was shown by PCR analysis with the primer pair, ASCL-gDNA-F and ASCL-gDNA-R, which bind to gDNA sequences located outside the locus-specific regions used for homologous recombination (Primers 1 and 2 in Fig 2A and 2B). Further verification of successful *PpASCL* allele replacement was obtained by using *PpASCL*- plus pTN182-specific PCR primer pairs to demonstrate the occurrence of both 5ʹ- and 3ʹ-targeted insertion (TI): ASCL-gDNA-F plus pTN182-5ʹ-R for 5ʹ TI and pTN182-3ʹ-F plus ASCL-gDNA-R for 3ʹ TI (Fig 2B).

For Southern blot analysis, *Acl*I-restricted gDNA (10 μg) was electrophoretically resolved using a 1.2% agarose gel, transferred to Amersham Hybond-N+ membrane (GE Healthcare, Mississauga, ON, Canada) and UV cross-linked to the membrane (UV Stratalinker, Stratagene, La Jolla, CA, USA). A 743 bp long *nptII*-specific probe was made by PCR using the primer pair, pTN-G418-F and pTN-G418-R (S1 Table), with pTN182. Probe digoxigenin (DIG)-labelling, Southern hybridization and immunological detection were performed using the DIG High Prime DNA Labeling and Detection Starter Kit II (Roche, Mannheim, Germany) according to the manufacturer’s instructions.

For reverse transcription (RT)-PCR, protonemal (100 mg), gametophoric (100 mg) and sporophytic tissues were ground to fine powder in liquid nitrogen with sterile mortar and pestle. For sporophytes, up to 1000 sporophytes (10–100 mg) from different developmental stages were harvested. Total RNA was extracted using an RNeasy Plant Mini kit (Qiagen), and each RNA sample (0.2–4 μg) was reverse transcribed using an Omniscript RT kit (Qiagen). Gene-specific and intron-spanning primers (S1 Table) were used to amplify corresponding cDNA molecules using a touchdown PCR program. The PCR program started at 95°C for 4 min, followed by 5 cycles of 94°C for 15 s, 65°C for 30 s and 70°C for 1.5 min. The annealing temperature was 60°C for the next 5 cycles, 55°C for a further 5 cycles and 50°C for the final 23 cycles. The use of intron-spanning primers provided assurance that amplicons had been derived from processed RNA transcripts and not from gDNA or unprocessed transcripts. *Physcomitrella actin3* (AY382283) was used as the internal reference.

### Phenotypic analysis of sporophytes

Gametangia were induced on two to four month old gametophytes grown in glass culture tubes by lowering the temperature to 16°C [30]. Fertilization and sporophyte formation were achieved by irrigating the gametophytes with 5 ml of sterile water containing 18 μM paba followed by further incubation at 16°C [31]. Developing sporophytes were visible within one week after irrigation. For observation of sporophytes, gametophores with sporophytes were removed from culture tubes and placed in sterile water in a Petri dish. Sporophytes were detached from the gametophores, counted and their developmental stage recorded. To release spores, sporophytes were gently ruptured in a drop of sterile water using fine forceps.

## Acetolysis

Acetolysis of spores was performed similarly to Dobritsa *et al*. [10]. Spore suspensions were centrifuged and decanted into a minimal volume of water. A large excess volume of freshly prepared 9:1 (v/v) acetic anhydride and sulphuric acid solution was added to the spores, which were then incubated at 70°C for 20 min. Treated spores were washed twice with sterile water and pipetted on to a slide. The slide was overlaid with a coverslip and the edges sealed with nail polish.

### Staining and light microscopy

Staining with Simplified Alexander’s stain, a stain used to distinguish between aborted and non-aborted spores [32], was performed according to Peterson *et al*. [33].

Sporophytes for cryosectioning were fixed with 4% paraformaldehyde overnight at 4°C. Fixed sporophytes were suspended in a tissue freezing medium (VWR International, Edmonton, AB, Canada), frozen on dry ice, and then mounted to microtome chuck. *pabB4* and *ascl-2* sporophytes were sectioned to a thickness of 20 μm and 30 μm respectively and placed on to positively charged slides. Sections were then stained with 1% toluidine blue O (TBO) and excess stain was removed with H_2_O. Images of slide mounted samples were taken with a Nikon Eclipse 80i compound light microscope equipped with a DS-Ri1 digital camera. All other light microscopy images were taken using a DS-Fi1 digital camera, mounted to a Nikon SMZ1500 stereoscopic microscope.

### Scanning electron microscopy

Untreated spores were fixed with formaldehyde and air dried on a coverslip overnight. Acetolysed spores were first washed with increasing concentrations of ethanol and then air dried on a coverslip overnight. The individual coverslips were mounted to SEM stubs with double-sided carbon tape and the samples were grounded with copper tape. They were then sputter-coated with gold for 2 min, with argon under vacuum, at a current of 7–8 mA (SC7620 Sputter Coater, Quorum Technologies, Guelph, ON, Canada). Gold coated samples were examined with the secondary electron detector fitted to a Jeol JSM-6360 scanning electron microscope. Samples were subjected to high vacuum during both sample preparation (gold-coating) and observation with the SEM. To determine the best conditions for operation, several spores were imaged at a range of beam currents (varied by increasing or decreasing the spot size), utilizing a smaller beam aperture and variable kV. Optimum conditions that provided the clearest and sharpest images at high magnification but that did not lead to sample charging or sample degradation were found to be 5 kV and a spot size of 25.

### Transmission electron microscopy

Spores were fixed in 2.5% glutaraldehyde in PBS solution for 30 min, washed and resuspended in 0.1 M sodium phosphate buffer, pH 7.4. Spores were pelleted by centrifugation and washed twice with the buffer. Spores were post-fixed with osmium tetroxide, washed three times with H_2_O and then pelleted. An equal volume of molten low melting point agarose was added, the spores resuspended and centrifuged again to pellet them in the tip of a microcentrifuge tube. Once the agarose had solidified, the embedded spores were fixed with the formaldehyde-glutaraldehyde mixture [34]. The tip was subsequently cut, and the spores were fixed a second time using a Pelco 3441 Laboratory Microwave System (Ted Pella, Redding, CA, USA). The fixation was carried out for 2 min at 100 W, stopped for 2 min, and repeated for another 2 min at 100 W. Fixed spores were decanted, then washed three times in H_2_O and cut into smaller pieces before dehydrating in a graded ethanol series (30, 40, 50, 70, 80, 90, 95, three times with 100%). A transition step of 1:1 ethanol:acetone was followed by a brief 100% acetone step prior to infiltration with Spurr’s epoxy resin. A dropwise method up to 25% resin in acetone was used, followed by microwave infiltration (18 in Hg vacuum, 3 min PL3) at 50% and 75% resin in acetone. One overnight step in 100% resin was followed by microwave infiltration (twice with 100% resin). Samples were embedded in flat molds and polymerized for 18 h at 65°C. Blocks were thin sectioned at 70 nm (Leica UC7) using a diamond knife (Diatome), mounted on uncoated 200 mesh copper grids and stained with 2% uranyl acetate and Reynold’s lead citrate for 12 and 5 min respectively. Imaging was performed at 80 kV with a Hitachi H7600 TEM.

## Results

### Molecular analysis of stable transformants

Stable transformants were tested by PCR for the occurrence of *PpASCL* allele replacement (Fig 2). This resulted in amplification of a larger DNA fragment (4319 bp) from the recombinant locus in the three stable transformants, *ascl1*–*3*, than from *PpASCL* in the untransformed control strain (2482 bp) (Fig 2C). Additional PCRs demonstrated both 5ʹ- and 3ʹ-TI in each of the three *ascl* transgenic strains. Thus, PCRs with ASCL-gDNA-F plus pTN182-5ʹ-R (for testing 5ʹ TI) and pTN182-3ʹ-F plus ASCL-gDNA-R (for testing 3ʹ TI) yielded amplification products of sizes consistent with those predicted, 1361 and 1188 bp respectively, when gDNA from *ascl* mutants was used (Fig 2B and 2D). Conversely, no DNA fragments were amplified from control gDNA. In summary, these data confirmed 249 bp of *PpASCL* coding sequence had been replaced in *ascl* mutants with the aminoglycoside resistance cassette from pTN182. Consequently, these transgenic strains should lack ASCL enzyme activity.

One *ascl* mutant, a*scl-2*, was also subjected to Southern hybridization to ascertain whether or not it possessed any ectopic copies of the transgenic DNA as a result of illegitimate recombination. Hybridization of *Acl*I-restricted *ascl-2* gDNA with *nptII*-specific DIG-labelled probe produced a discrete Southern signal corresponding to a single, approx. 6 kb DNA fragment, which accorded closely with the predicted size, 5991 bp, of the expected *Acl*I restriction fragment containing a recombinant, i.e. mutant, *PpASCL* allele. No signal was detected with the control gDNA (S1 Fig).

**Fig 3:**
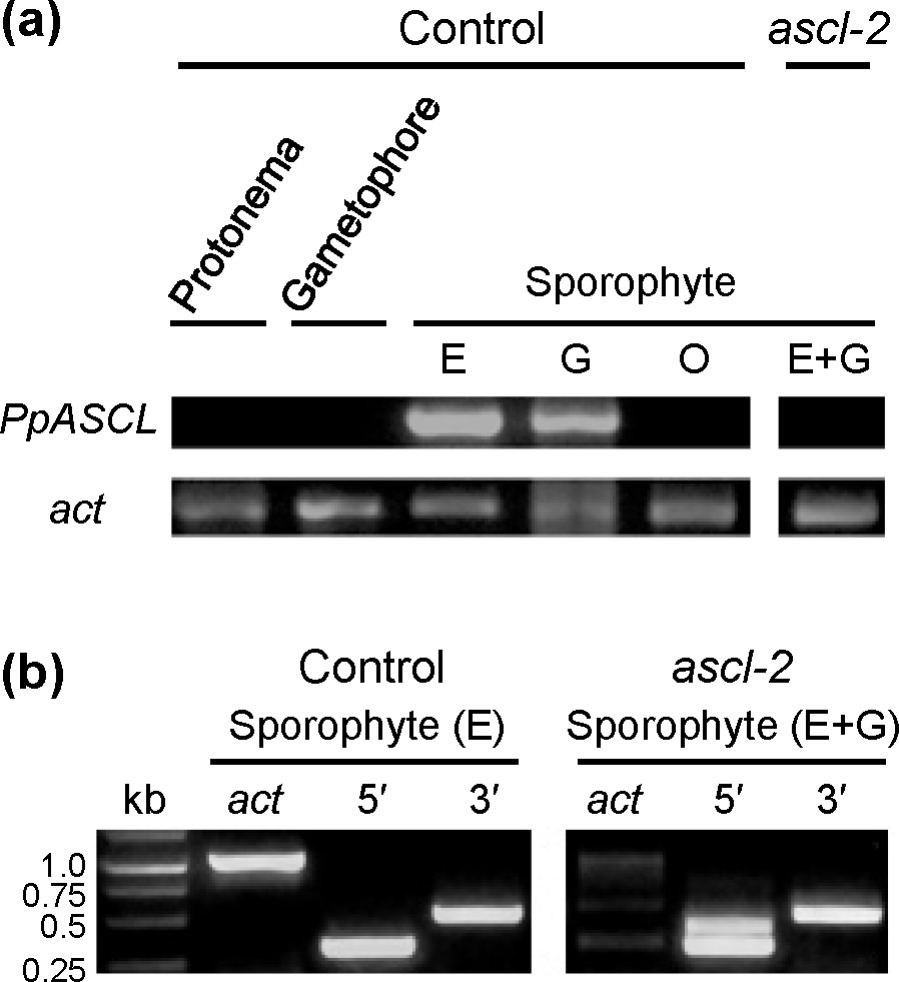
RT-PCR analysis of (a) *PpASCL* expression in *Physcomitrella* at successive developmental stages and (b) *PpASCL* promoter activity in *ascl-2*. (a) The primers, ASCL-RT-F and ASCL-RT-R (S1 Table), were used to amplify 1115 nucleotides, including 1108 nts from the protein coding region of the *PpASCL* transcript. In the untransformed *pabB4* control, *PpASCL* expression was detected in sporophytes at the expanding (E) and green (G) capsule stages, but not in the mature orange (O) stage nor in protonemata and gametophores. RT-PCR using *ascl-2* sporophytes, comprising a minority at the expanding and a majority at the green capsule stages respectively (E+G), failed to yield the 1115 bp amplicon thus providing additional evidence that *PpASCL* has been knocked out in this mutant. (b) *PpASCL* promoter activity was examined in *pabB4* E sporophytes and in *ascl-2* E+G sporophytes. The primer pair, ASCL-RT-F and 5'R-ASCL-ClaI (S1 Table), was used to amplify a 5′ region predicted to be 332 nucleotides long in mature *PpASCL* transcripts. Similarly, the primer pair, 3'F-ASCL-NdeI and ASCL-RT-F, was used to amplify a 3′ region predicted to be 536 nucleotides long in mature *PpASCL* transcripts. Both expected amplicons were generated with sporophytes of both strains. An additional 5ʹ amplicon (upper band) was produced with *ascl-2* that, based on its size, we attribute to inefficient splicing of intron 1 from the mutant pre-mRNA. Expression of the *Physcomitrella actin3* gene (*act*) was used as a reference.

RT-PCR was used to demonstrate that *PpASCL* is expressed in a tissue- and developmental stage-specific manner. It was highly expressed in control sporophytes at the “expanding capsule” (E) and “green” (G) developmental stages but no expression was detected in “orange” sporophytes (O), protonemata or gametophores (Fig 3A). Failure to produce the 1115 bp amplicon when RT-PCR was performed using *ascl-2* E + G sporophytes provided further confirmation that *PpASCL* had been successfully knocked out in this mutant. To test whether the *PpASCL* promoter is under the same spatio-temporal regulation in the control and *ascl-2*, we performed additional RT-PCRs with *pabB4* E sporophytes (*left panel*, Fig 3B) and *ascl-2* E + G sporophytes (*right panel*, Fig 3B), targeting separately the 3' and 5' ends of their respective normal and mutant *PpASCL* transcripts. The primer pair designed to amplify a 3′ region common to both transcripts yielded the predicted 536 bp amplicon in each case. Similarly, the primer pair targeting a 5′ region common to both transcripts produced the expected 332 bp amplicon. However, in the latter case, a larger amplicon was also generated in *ascl-2* (lane 5′, *right*, Fig 3B). The size of this extra amplicon is consistent with its including the 121 nt long intron present towards the 5ʹ end of unprocessed pre-mRNA (intron 1 of *PpASCL*). Since the 3ʹ end of this intron is only 63 nucleotides upstream from the portion of *PpASCL* replaced by a selection cassette in *ascl-2*, a plausible explanation is that a splicing enhancer was located in the section of *PpASCL* replaced during gene knockout and its loss has resulted in less efficient removal of intron 1. It is unlikely to have resulted from contaminating gDNA since, if that had been the case, a larger amplicon incorporating intron 2 (107 nts long) would have been produced with the other primer pair. Also, no larger amplicons were generated from control sporophytes subjected to the same experimental procedures. Thus, transcriptional regulation appears to be identical for the normal and mutant *PpASCL* alleles.

Collectively, these observations indicated that *ascl-2* was a knocked out transgenic *PpASCL* mutant, which had resulted from single-copy allele replacement unaccompanied by insertion of ectopic copies of the replacement vector.

*Ascl-2* became the strain of choice for further experiments.

### The developmental phenotype of *ascl-2*

Untransformed *pabB4* control and *ascl-2* gametophytes appeared to be morphologically and developmentally identical (S2 Fig). The formation of gametangia and fertilization were unaffected in *ascl-2* as were the early stages of sporophytic morphogenesis. However, during late stages of sporophytic maturation, there were marked differences between these strains.

### Morphogenesis of *ascl-2* sporophytes

In this study, the sporophytic developmental stages are defined according to the sporophytic maturation and general colouration of the spores [35] as the initial development, expanding capsule, green, yellow and orange stages. Detailed descriptions of *pabB4* sporophytes at different developmental stages are provided in S2 Table.

**Fig 4:**
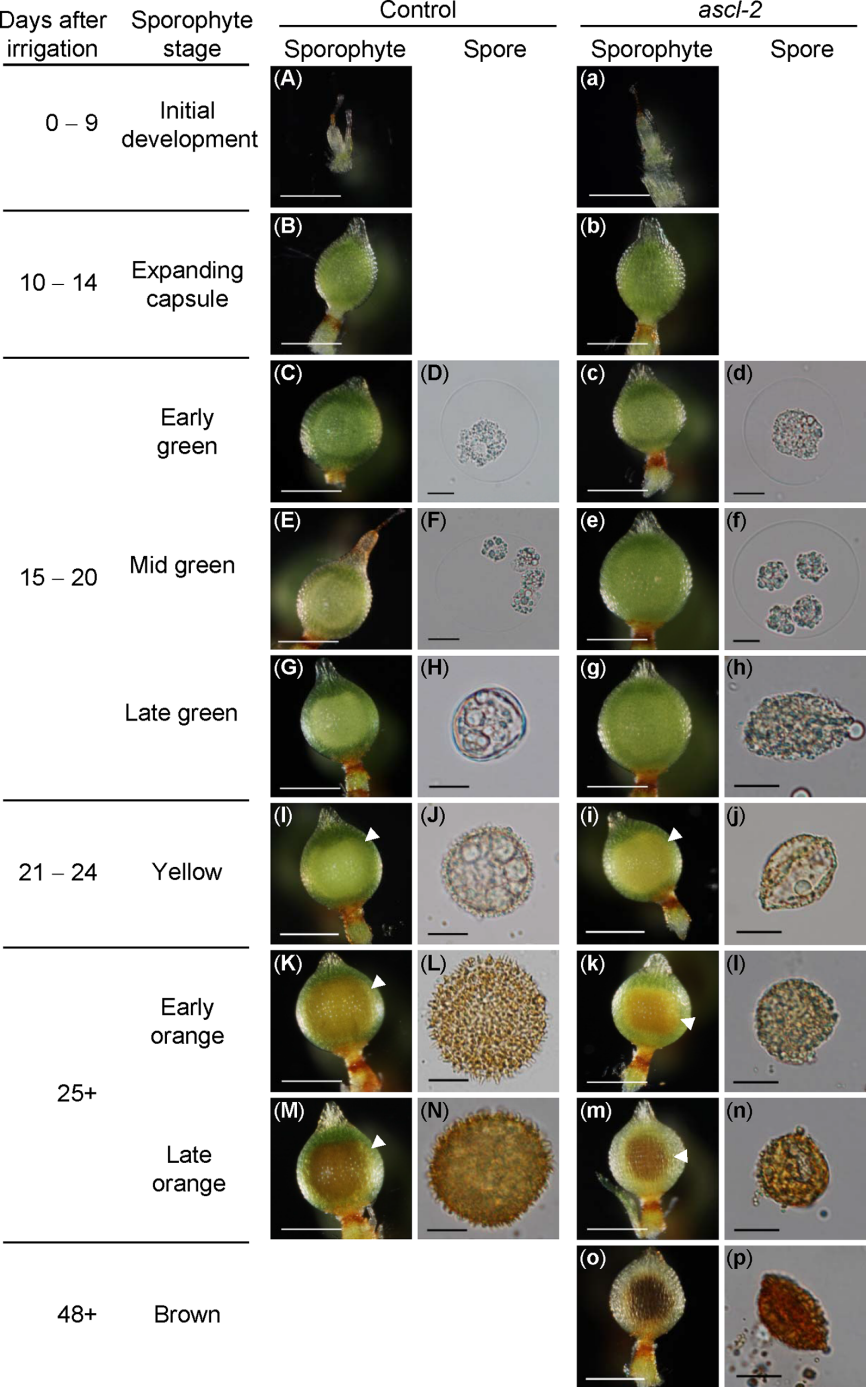
Developmental timelines for *pabB4*, the untransformed control strain, and *ascl-2* sporophytes and spores. Photomicrographs of the typical morphologies of sporophytes and spores of control (A–N) and *ascl-2* (a–p) at successive developmental stages. The number of days after irrigation of the cultures is shown with the names assigned to each sporophytic stage. Some stages have been subdivided to allow more detailed description of changes in spore development. White arrowheads denote the outlines of the spore masses within capsules. No spores are seen during the initial growth and expanding capsule stages. The control did not reach the brown sporophytic stage during the observation period. Sporophyte scale bars = 500 μm; Spore scale bars = 10 μm.

Growth and morphogenesis of *ascl-2* and *pabB4* control sporophytes were indistinguishable up to and including the mid green stage (Fig 4A–4F, 4a–4f). The earliest differences between control and *ascl-2* sporophytes appeared at the late green stage of development (Fig 4G and 4H,4g,4h). In *ascl-2*, the developing spore mass exhibited a less well defined outline and did not fill the space within the capsule wall to the same extent as in the control (Fig 4I, 4K and 4M,4i,4k,4m, white arrowheads). This appeared to be due to a difference in size of the spores of the respective strains. Whereas control spores increased in size as they matured, *ascl-2* spores did not. The general colouration of the spores did not appear to be different in the two strains (Fig 4K–4N, 4k–4n). While the overall developmental timing of *ascl-2* sporophytic stages coincided with that of the control, a unique brown stage was observed in *ascl-2*, which formed from a subset of orange sporophytes after day 48 (Fig 4O and 4P).

Free spores were almost never released from *ascl-2* sporophytes. Disintegration of the outer walls of aged, orange and brown, *ascl-2* capsules typically did not result in spore release. Some of these capsules contained an amorphous material that caused the spores to aggregate and prevented their dispersal. A similar sticky extracellular material has been described in Arabidopsis *ASCL* and *TETRAKETIDE α-PYRONE REDUCTASE* mutant anthers [13, 14, 36, 37].

**Fig 5:**
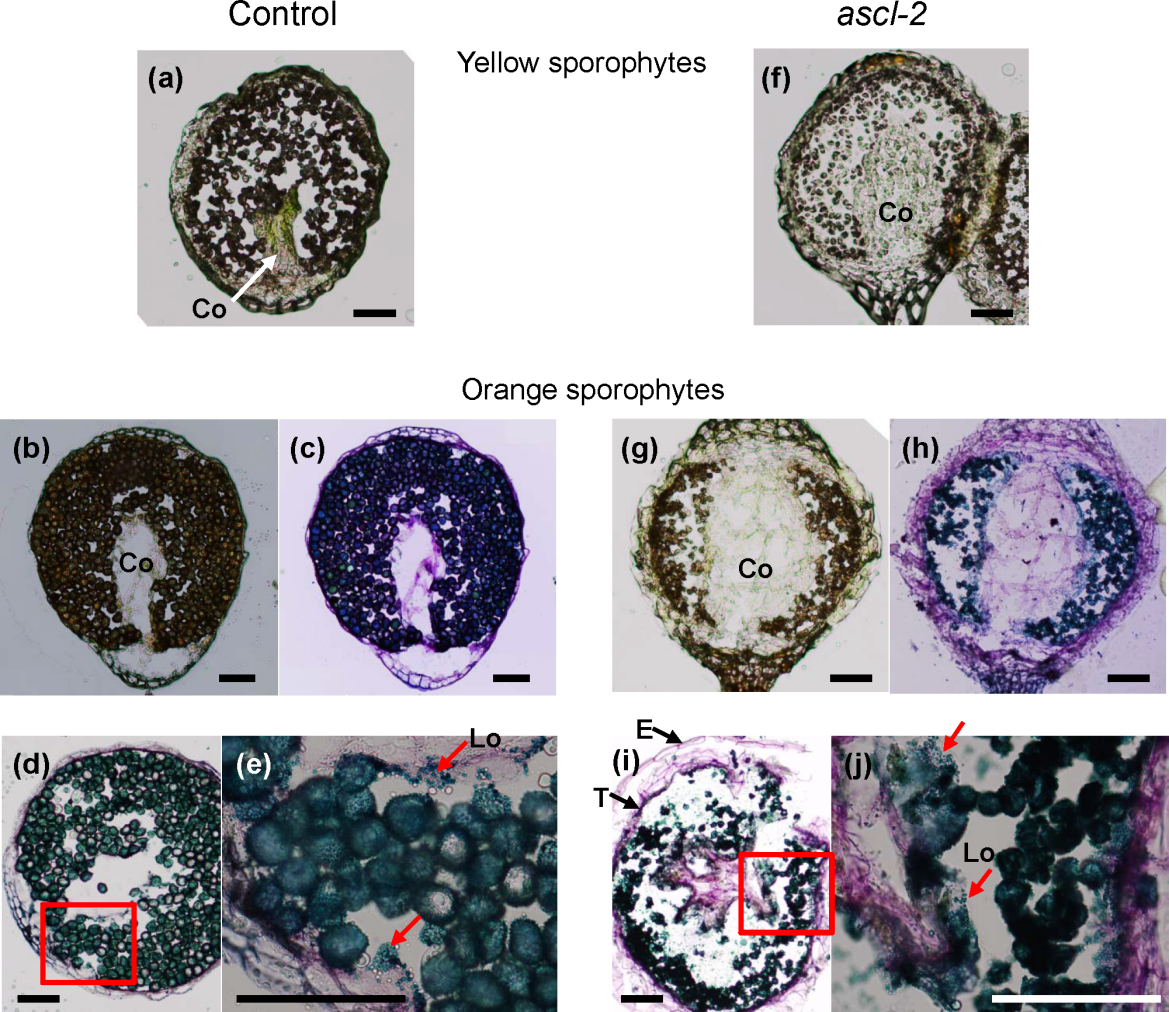
Photomicrographs of cryosectioned *pabB4* control and *ascl-2* sporophytes. Cross sections of control (20 μm, a–e) and *ascl-2* (30 μm, f–j) sporophytes were taken at the yellow (a,f) and orange (b–e, g–j) stages. Images were saved before (a,b,f,g) and after toluidine blue O staining (c–e, h–j). Orbicules present in locules of the air-space and on the tapetum wall surface are indicated with red arrows in (e) and (j). Sections (e) and (j) are magnified images of red-boxed areas of (d) and (i), respectively. Co, columella; E, epidermis; Lo, locule; T, tapetum. Scale bars = 100 μm.

Further differences were revealed when cross sections of control and *ascl-2* sporophytes were compared (Fig 5). Firstly, while mature spores filled the locules comprising the air-space of yellow and orange control sporophytes (Fig 5A, 5B and 5D), the smaller spores of *ascl-2* aggregated, leaving much of the air-space empty (Fig 5F, 5G and 5I). Secondly, the central, column-shaped columella of control capsules atrophied by the yellow stage and became noticeably smaller than in *ascl-2* capsules in which it filled much of the inner capsular space from apex to base (Fig 5B and 5G). Thirdly, a tapetal layer, which stained darkly and formed a lining around the air-space, was more prominent in *ascl-2* capsules (Fig 5I and 5J). Lastly, in *ascl-2* capsules, sporopollenin orbicules (stained blue) were present around spores throughout the locules of the air-space and next to the tapetal layers, while the distribution of those in control capsules appeared to be more localized and mainly next to the tapetum (Fig 5E and 5J).

#### Spore morphology

**Fig 6:**
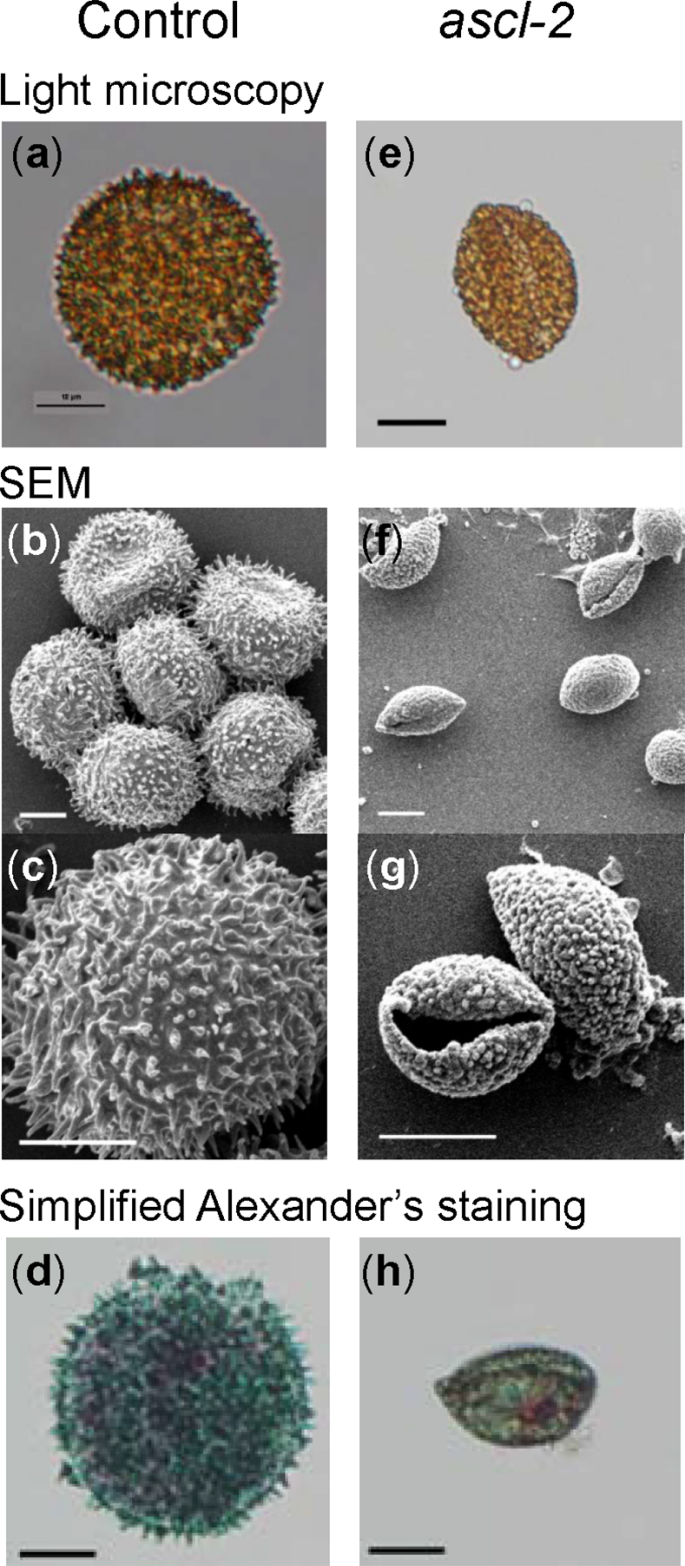
Morphological comparison of orange stage *pabB4* control and *ascl-2* spores. Images of typical spores isolated from mature orange sporophytes were acquired using light microscopy (a,e) and SEM (b,c,f,g). Spores from the control (a–c) and *ascl-2* (e–g) are shown. Light microscopy images of control (d) and *ascl-2* (h) spores after treatment with simplified Alexander’s stain are also shown. Scale bars = 10 μm.

Control spores are spherical with an echinate surface composed of perine (perispore) elements, each with a tapering end and taller than 1 μm [38]. The abnormal phenotype of *ascl-2* spores was apparent in the free microspores produced by dissociation of tetrads of meiospores and present at the late green sporophytic developmental stage (Fig 4H). For meaningful comparison of spores from different strains, spores were isolated in all cases from the orange sporophytic stage. All three stable *ascl* mutants made spores, which were smaller than those of the untransformed control and had a distinctive, abnormal morphology (Fig 6 and S3 Fig). The cell wall of mutant spores appeared to have collapsed resulting in many of the spores exhibiting a biconvex outline. The diameter of control spores, excluding ornamentation, was 28 ± 2.0 μm (*n* = 132, mean ± S.D.), whereas the longest dimension of *ascl-2* spores was 18 ± 1.9 μm (*n* = 146). The mutant spores had a highly irregular surface with globular, granulose protrusions, and lacked the characteristic perine spines found on the spore wall of the control. The morphological differences were clearest in SEM images, which revealed that the typical perine spines of the control were replaced with granulose protrusions (Fig 6G and S3J–S3L Fig). Interestingly, SEM revealed an end-to-end fissure in *ascl* spore walls, which was also observed occasionally with light microscopy (S3B–S3D Fig). Fissuring does not appear to be an artefact of the SEM imaging process since it was not observed in the SEM images of control spores obtained under the identical conditions (Fig 6B and S3E Fig).

**Fig 7:**
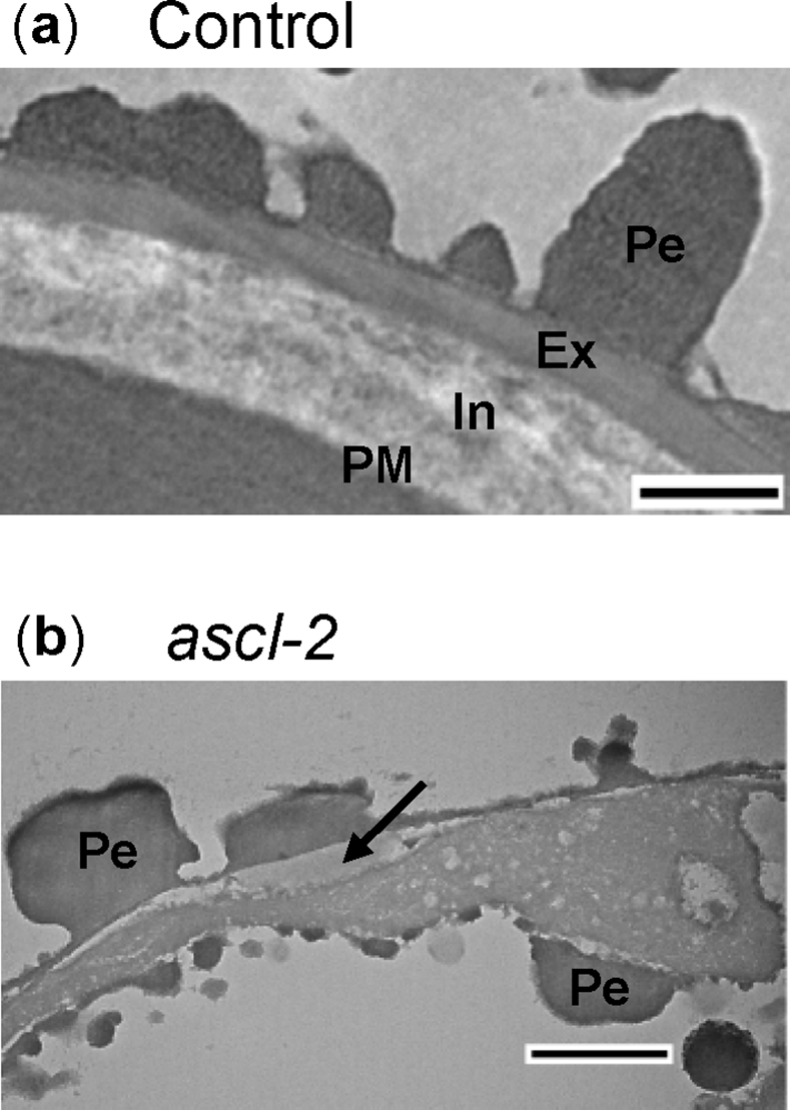
Transmission electron micrographs of *pabB4* control and ascl-2 spores. Cross sections of spores from mature orange control (a) and *ascl-2* (b) sporophytes were examined with TEM. An amorphous layer found below much of the perine is indicated by an arrow in (b). Ex, exine; In, intine; Pe, perine; PM, plasma membrane. Scale bars = 500 nm.

TEM of control spores clearly depicted the typical stratification of the spore wall comprising the innermost, fibrillar intine, a more electron dense exine (exospore) and the outermost perine layer with spines (Fig 7A) [39]. In *ascl-2* spores, the only seemingly identifiable layer appeared to be the perine surrounding the spore and comprising the abnormal ornamentation on its surface (Fig 7B). An irregular, amorphous layer was present below much of the outer perine that may represent remnants of the intine (or exine) acting as the foundation for perine deposition (indicated by an arrow in Fig 7B). As mentioned above, the integrity of the spore wall in *ascl-2* was compromised by an opening or fissure in the wall and the *ascl-2* spores were only partially filled with cytoplasm, presumably because of leakage of cytoplasm from the spores via the fissure in the mutant spore wall. There also appeared to be uneven deposition of perine or perine-like material at the surface of the remaining cytoplasm within the *ascl-2* spores (Fig 7B).

**Fig 8:**
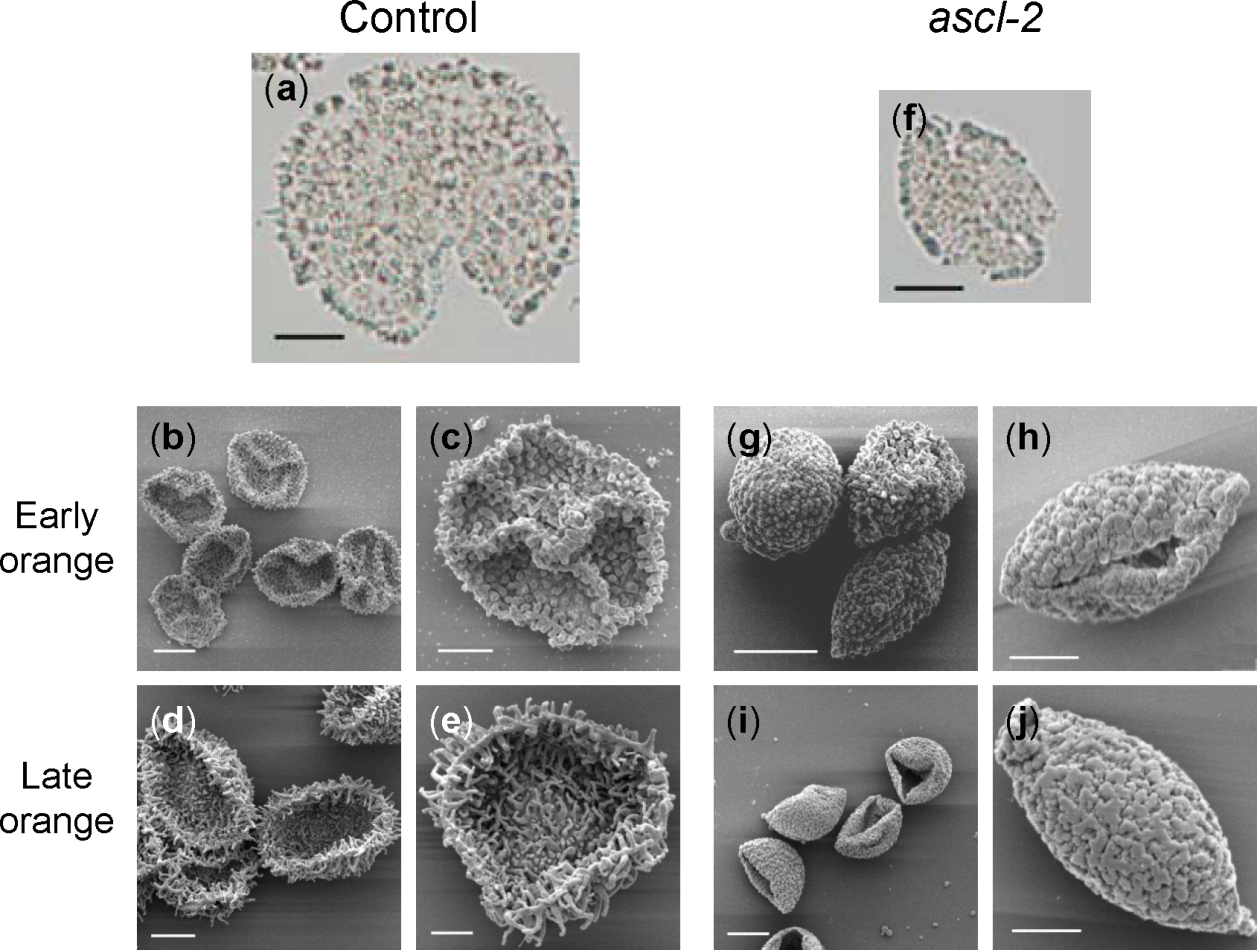
Light microscopy and SEM images of acetolysed spores. Photomicrographs of spores of *pabB4* control (a) and *ascl-2* (f) and SEM images of gold coated spores from control (b–e) and *ascl-2* (g–j) after acetolysis. Spores were isolated from the orange sporophytic developmental stage. Triradiate ridges are visible in some collapsed control spores (b,c). Scale bars (a,b,d,f,g,i) = 10 μm; (c,e,h,j) = 5 μm.

Acetolysis showed that both control and *ascl-2* spore walls are resistant to this chemical treatment. After acetolysis, a slightly yellow-coloured remnant of the spore wall remained intact, comprising the spines of the control and the irregular surface ornamentation of *ascl-2* (Fig 8A and 8F). SEM images of acetolysed spores from the early and late orange sporophytic stages revealed the intact echinate and granulose surface patterns of control and *ascl-2* spores, respectively (Fig 8E and 8J). Untreated control spores, observed by SEM with and without fixation, were comparable, with none of the spores collapsing (Fig 6B and 6C). By contrast, acetolysed, unfixed control spores collapsed in the vacuum of the SEM (Fig 8B–8E), while those of *ascl-2*, which had a compromised and fractured cell wall, were unaffected (Fig 8G–8J). In some cases, the collapsed control spores exhibited a triradiate ridge indicative of their tetrad origin (Fig 8B and 8C).

#### Spore viability

*PabB4* control spores from the yellow and orange sporophytic stages, plated on solid ABC medium supplemented with paba and incubated in continuous white light, began germinating after 4 days. By the fourth day, a large percentage of orange spores had germinated and most of them had germinated after 7 days. Under identical conditions, no *ascl-2* spores from any stage of sporophytic development had germinated. Simplified Alexander’s stain revealed that cytoplasm uniformly filled most of the interior of control spores whereas the majority of stained *ascl-2* spores exhibited a comparatively irregular distribution of cytoplasm (Fig 6D and 6H). Thus, compared to control spores, *ascl-2* spores were depleted in cytoplasm and were nonviable.

## Discussion

### Evolutionarily conserved genes involved in exine formation

The *Physcomitrella* genome contains orthologues of Arabidopsis genes involved in exine formation, more specifically in the biosynthesis of sporopollenin. These moss orthologues were first identified by phylogenetic and EST abundance analyses [9, 21, 22], and their expression in *Physcomitrella* spores was later supported by a genome-wide microarray expression study [20]. Also, they were shown to be up-regulated in early stage sporophytes compared to gametophytes [40]. However, direct evidence of the involvement of the moss orthologues in spore wall formation was initially lacking. Ara *et al*. reported that PpCYP703B2 partially rescued a defect in the exine layer of the pollen wall of the rice mutant, *cyp703a3* [41]. When the *Physcomitrella* orthologue encoding GAMYB, a transciption factor that regulates *CYP703* expression, was knocked out, the resulting *Ppgamyb2* spores had severely defective perine and outer exine layers [41]. These results are in agreement with the proposed role of CYP703B2 in sporopollenin biosynthesis [9, 21]. More importantly, Wallace *et al*. [8] created knockout lines of *PpMS2-1*, a *Physcomitrella* orthologue of Arabidopsis *MS2*, and showed that *PpMS2-1* is essential for normal spore wall development, providing the first *in planta* evidence for conserved functions of Arabidopsis and *Physcomitrella* orthologues implicated in sporopollenin biosynthesis. Our study shows that *PpASCL*, the putative *Physcomitrella* orthologue of Arabidopsis *PKSA* and *PKSB* [21], is required for proper exine and intine formation and also for the construction of a normal perine layer. The abnormal perine projections of *ascl-2* and *Ppms2-1* spores are similar in appearance and, in both cases, the distinction between intine and exine layers has been lost or blurred and the combined width of these two layers has been reduced when compared to the trilaminate spore wall of control strains. Interestingly, spores of both mutants are smaller than control spores. These combined observations strengthen the contention that sporopollenin biosynthesis is an ancient adaptation to conditions in a terrestrial environment that has been conserved throughout subsequent land plant evolution.

### The *Physcomitrella ascl* mutant phenotype is both similar to and distinct from that of the Arabidopsis *pksa pksb* double mutant

In *Physcomitrella* and other mosses belonging to the Bryopsida class, exine formation and sporopollenin deposition are thought to occur during the tetrad stage of spore development [22]. Consistent with this, *PpASCL* expression was detected exclusively in the early stages of spore development. Similarly, expression of Arabidopsis *PKSA* and *PKSB* is tightly regulated both temporally and spatially, occurring primarily during the tetrad and/or free microspore stages of pollen development [14].

ASCL is a hydroxyalkyl α-pyrone synthase *in vitro* and hydroxyalkyl α-pyrones are presumed to be *in planta* sporopollenin precursors. Based on spectroscopic data, Fraser *et al*. [42] proposed that the structure of sporopollenin is the same in all land plants. Sporopollenin is the major constituent of the exine layer of both spore and pollen walls. Both *ascl-2* spores and *pksa pksb* pollen lack a recognisable exine layer and are inviable. However, the abnormal phenotype of *pksa pksb* double mutants in Arabidopsis is more severe than that of *ascl* single mutants in *Physcomitrella*. The majority of *pksa pksb* double mutants fail to produce pollen grains apparently because their microspores atrophy [14]. This suggests that Arabidopsis is unable to produce robust, albeit defective, sporopollenin without ASCL products. Conversely, degeneration of defective *ascl-2* spores did not occur and aborted spores remained intact even after several months. While bryophytes have a single-copy *ASCL*, angiosperm species possess at least two *PKS* paralogues, which form separate clades in phylogenetic trees, suggesting functional diversification [15]. Collectively, these results indicate that ASCL products are components of sporopollenin in bryophytes and angiosperms but their contributions to sporopollenin integrity in these two plant groups may be different. Arabidopsis *PKSA* and *PKSB* and their products (presumed to be tri- and tetraketide α-pyrones) appear to have a more integral role in exine formation than does *Physcomitrella PpASCL*. This may result partly from differences between spores and pollen with respect to the process of exine formation, especially the mode of sporopollenin deposition, during wall formation [8, 22].

It is not known which sporophytic cells express *PpASCL*. Most characterized angiosperm sporopollenin monomer biosynthetic genes are preferentially expressed in anther tapetal cells and a few are also expressed in developing microspores. CYP703A2, an in-chain hydroxylase, is present in the tapetum and developing microspores [9]. *Cyp703a2* mutant pollen development is indistinguishable from that of the wild type until after release from the tetrad when two sizes of pollen are observed, one of similar size to wild type and another much smaller that appears to have been arrested in development soon after free microspore release [9]. *PKSA* and *PKSB* are also expressed in the tetrads of developing microspores but neither single nor double mutants exhibit a reduced pollen size, possibly because of the observed minimal tetrad gene expression [14]. The smaller size of *ascl-2* and *Ppms2-1* spores and *cyp703a2* pollen is atypical. Most other mutants containing mutated sporopollenin biosynthesis genes produce normal-sized mature pollen grains. Smaller *ascl-2* spore size may be the result of premature arrest of spore development and perhaps indicates earlier expression of *PpASCL* in spore development compared to the time of expression of most sporopollenin biosynthesis genes in Arabidopsis pollen.

Alexander’s or Simplified Alexander’s stain has been used to visualize the differences between aborted and non-aborted pollen [32, 33]. *NtPKS1i*, a *Nicotiana tabacum* RNAi line with knocked down expression of *NtPKS1*, a *PpASCL* ortholog, is male sterile. Pollen grains from mature *NtPKS1i* anthers do not contain cytoplasm as demonstrated by Alexander’s staining. Wang *et al*. [43] suggested that loss of the cytoplasm results from cytoplasmic leakage through a structurally compromised cell wall during microspore development. Similarly, *ascl-2* spores are non-viable and Simplified Alexander’s staining revealed that the majority of them exhibit an irregular distribution of cytoplasm. The wall of *ascl-2* spores may be compromised in a similar way to that of *NtPKS1i* pollen, early in free microspore development, causing developmental arrest and abortion of the spores from cytoplasmic leakage.

Since intine, which is substantially comprised of pectin, appears to be absent or greatly reduced in the wall of *ascl-2* spores, we cannot be certain that PpASCL is involved exclusively in sporopollenin synthesis. However, we suggest that the most probable explanation of the lack of intine in this case is that the development of *ascl* spores is arrested prior to intine deposition as a result of compromised sporopollenin synthesis.

### Defective sporopollenin is resistant to acetolysis

Acetolysis treatment is used to determine chemical resistance of the sporopollenin, and defects thereof, in spore and pollen walls. Acetolysis experiments with pollen from Arabidopsis strains mutated in *MS2*, *CYP704B1*, *PKSA* and *PKSB* reveal various levels of chemical sensitivity. *MS2* pollen grains are moderately sensitive to acetolysis since most of them are lysed but retain remnants of pollen cell wall after this treatment [44]. Pollen grains from a cytochrome P450 mutant, *cyp704b1*, lack a distinguishable exine layer and are destroyed by acetolysis [10]. Single mutants of *PKSA* (*LAP6*) and *PKSB* (*LAP5*) produce pollen that are resistant to acetolysis, retaining their shape and overall structure [37]. The *ascl-2* spore wall is as resistant to acetolysis as that of the control with the distinct spore morphologies of these strains being unaffected. Specifically, the spines of the control and the irregular granula of *ascl-2* spores are readily discernible in SEM images of acetolysed spores. This indicates that, despite the structural integrity and organization of the *ascl-2* spore cell wall being compromised, it contains a defective sporopollenin with chemical resistance equivalent to that of control sporopollenin. Similar resistance of a defective sporopollenin to acetolysis was observed in the *Physcomitrella MS2* knockout line [8]. Such observations oblige a cautious interpretation of the level of acetolysis resistance as a means of assessing the condition of sporopollenin.

The structure of sporopollenin is poorly characterized compared to that of other biopolymers mainly due to its resistance to chemical and biochemical treatments. In particular, the chemical nature of *in planta* ASCL products and their contributions to sporopollenin are unclear. Studying the defective sporopollenin of *ascl-2* and other knockout lines of sporopollenin biosynthesis genes should provide clarification.

In conclusion, the abnormal phenotype of *ascl-2*, an *ASCL* knockout mutant in the moss, *Physcomitrella patens*, is consistent with a role for PpASCL in exine and perine formation during spore development and provides additional support for an ancient, evolutionarily conserved pathway of sporopollenin biosynthesis. Finally, it seems reasonable to speculate that some or perhaps all the anatomical abnormalities of *ascl* sporophytes result secondarily from the early arrestment of spore development in *ascl* mutants.

## Supporting Information

S1 Fig Southern blot analysis of *ascl-2*. Southern blot of *Acl*I restricted *pabB4* control and *ascl-2* gDNA, hybridized with a DIG-labelled probe specific to the *npt-II* resistance cassette. Probe hybridization is denoted by an arrowhead. The positions of DNA size markers are indicated at left. The expected size of the *Acl*I restriction fragment from a recombinant, i.e. mutant, *PpASCL* allele is 5991 bp.
(PDF)

S2 Fig Photomicrographs of *pabB4* control and *ascl-2* gametophytes. Six weeks old, vegetatively propagated colonies of *pabB4* and *ascl-2*. Scale bars = 5 mm.
(PDF)

S3 Fig Morphologies of control (*pabB4*) and *ascl* spores. (a–d) Photomicrographs of spores isolated from mature sporophytes. Fractured *ascl* spore walls are clearly visible in (b) and (c). *Ascl* plants produced smaller spores. The diameter of control spores was 28 ± 2.0 μm (*n* = 132, mean ± S.D.); the longest dimension of *ascl* spores was 20 ± 1.7 μm (*n* = 63), 18 ± 1.9 (*n* = 146) and 19 ± 2.2 (*n* = 39) in *ascl-1*, *ascl-2* and *ascl-3*, respectively. The *ascl* spores were significantly smaller than the control spores (ANOVA, p < 0.0001). (e–l) SEM images of fixed, gold-coated spores. All scale bars = 10 μm.
(PDF)

S4 Fig TEM images of control (*pabB4*) and *ascl-2* spores. Cross sections of control (a) and *ascl-2* (b,c) spores from orange sporophytes were examined by TEM. In the control, the spore wall is intact and the spore is filled with cytoplasm, which here shows little structural resolution except for the faint outlines of oil droplets. The integrity of the spore wall in *ascl-2* has been compromised and the arrows in (b) and (c) indicate the position of an opening or fissure in the wall. The *ascl-2* spores are only partially filled with cytoplasm, presumably because of leakage of cytoplasm from the spores via the fissure in the mutant spore wall. There also appears to be uneven deposition of perine or perine-like material at the surface of the remaining cytoplasm within the *ascl-2* spores. The perine projections on the outside of *ascl-2* spores are smoother and less pointed than those on the outside of control spores. Magnified images of areas outlined in red in (a) and (c) are shown in Fig 7A and 7B, respectively. Scale bars = 2 μm.
(PDF)

S1 Table Primer sequences. (PDF)

S2 Table Descriptions of developmental stages of sporophytes of *pabB4*, the untransformed control strain. (PDF)

S3 Table Raw data of sporophytic development. The number of sporophytes at each developmental stage is given as a percentage of the total number in a culture tube.
(PDF)
