# Supplementary material for: PpASCL, the Physcomitrella patens Anther-Specific Chalcone Synthase-Like Enzyme Implicated in Sporopollenin Biosynthesis, Is Needed for Integrity of the Moss Spore Wall and Spore Viability
Source: PLoS One. 2016 Jan 11;11(1):e0146817. doi: 10.1371/journal.pone.0146817 (PMC4709238; doi:10.1371/journal.pone.0146817)

S1 Fig. Southern blot analysis of *ascl-2*.
Southern blot of *Acl*I restricted *pabB4* control and *ascl-2* gDNA, hybridized with a DIG-labelled probe specific to the *npt-II* resistance cassette. Probe hybridization is denoted by an arrowhead. The positions of DNA size markers are indicated at left. The expected size of the *Acl*I restriction fragment from a recombinant, i.e. mutant, *PpASCL* allele is 5991 bp.

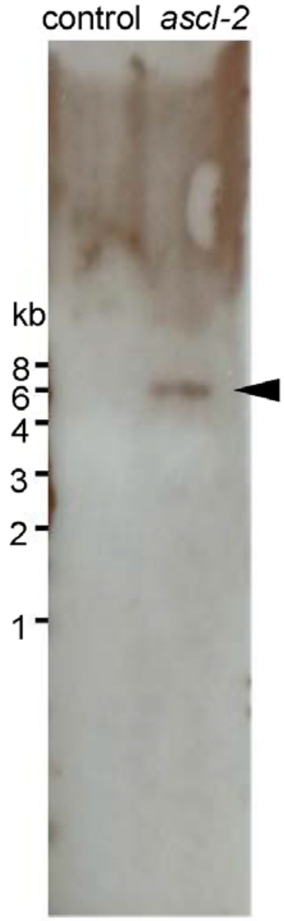

Supplement: S1 Fig — Southern blot of AclI restricted pabB4 control and ascl-2 gDNA, hybridized with a DIG-labelled probe specific to the npt-II resistance cassette. Probe hybridization is denoted by an arrowhead. The positions of DNA size markers are indicated at left. The expected size of the AclI restriction fragment from a recombinant, i.e. mutant, PpASCL allele is 5991 bp. (PDF) [file pone.0146817.s001.pdf]
