# Supplementary material for: PpASCL, the Physcomitrella patens Anther-Specific Chalcone Synthase-Like Enzyme Implicated in Sporopollenin Biosynthesis, Is Needed for Integrity of the Moss Spore Wall and Spore Viability
Source: PLoS One. 2016 Jan 11;11(1):e0146817. doi: 10.1371/journal.pone.0146817 (PMC4709238; doi:10.1371/journal.pone.0146817)

S2 Fig. Photomicrographs of *pabB4* control and *ascl-2* gametophytes.
Six weeks old, vegetatively propagated colonies of *pabB4* and *ascl-2*. Scale bars = 5 mm.

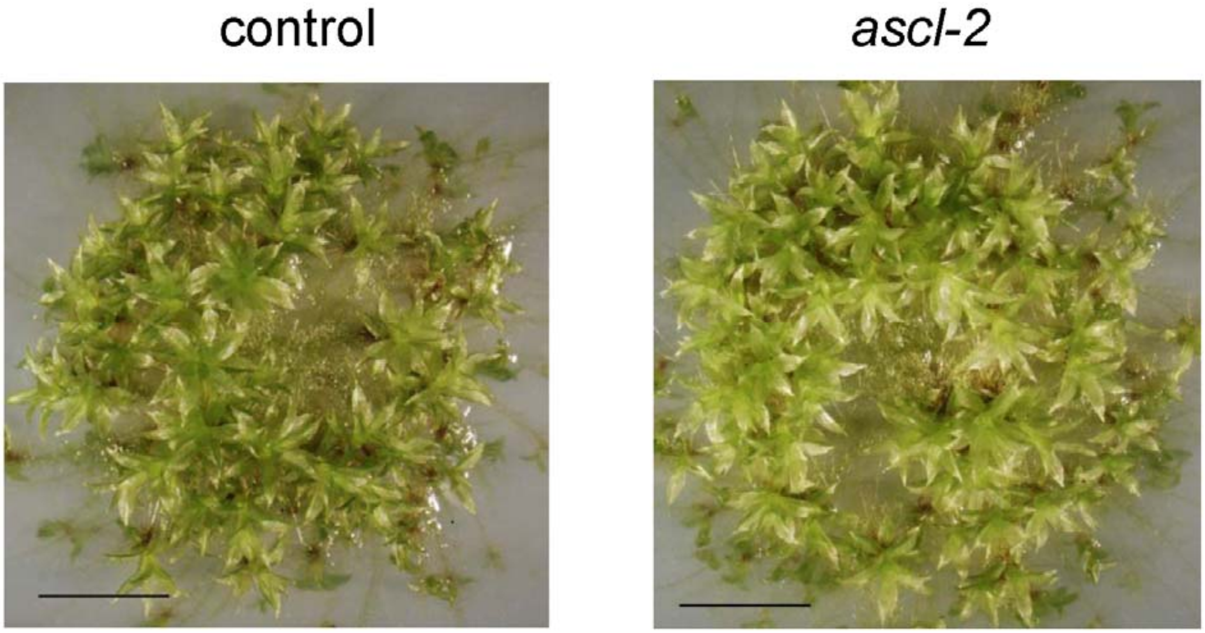

Supplement: S2 Fig — Six weeks old, vegetatively propagated colonies of pabB4 and ascl-2. Scale bars = 5 mm. (PDF) [file pone.0146817.s002.pdf]
