# Supplementary material for: PpASCL, the Physcomitrella patens Anther-Specific Chalcone Synthase-Like Enzyme Implicated in Sporopollenin Biosynthesis, Is Needed for Integrity of the Moss Spore Wall and Spore Viability
Source: PLoS One. 2016 Jan 11;11(1):e0146817. doi: 10.1371/journal.pone.0146817 (PMC4709238; doi:10.1371/journal.pone.0146817)

S3 Fig. Morphologies of control (*pabB4*) and *ascl* spores.

(a–d) Photomicrographs of spores isolated from mature sporophytes. Fractured *ascl* spore walls are clearly visible in (b) and (c). *Ascl* plants produced smaller spores. The diameter of control spores was 28 ± 2.0 µm (*n* = 132, mean ± S.D.); the longest dimension of *ascl* spores was 20 ± 1.7 µm (*n* = 63), 18 ± 1.9 (*n* = 146) and 19 ± 2.2 (*n* = 39) in *ascl-1*, *ascl-2* and *ascl-3*, respectively. The *ascl* spores were significantly smaller than the control spores (ANOVA, $p < 0.0001$). (e–l) SEM images of fixed, gold-coated spores. All scale bars = 10 µm.

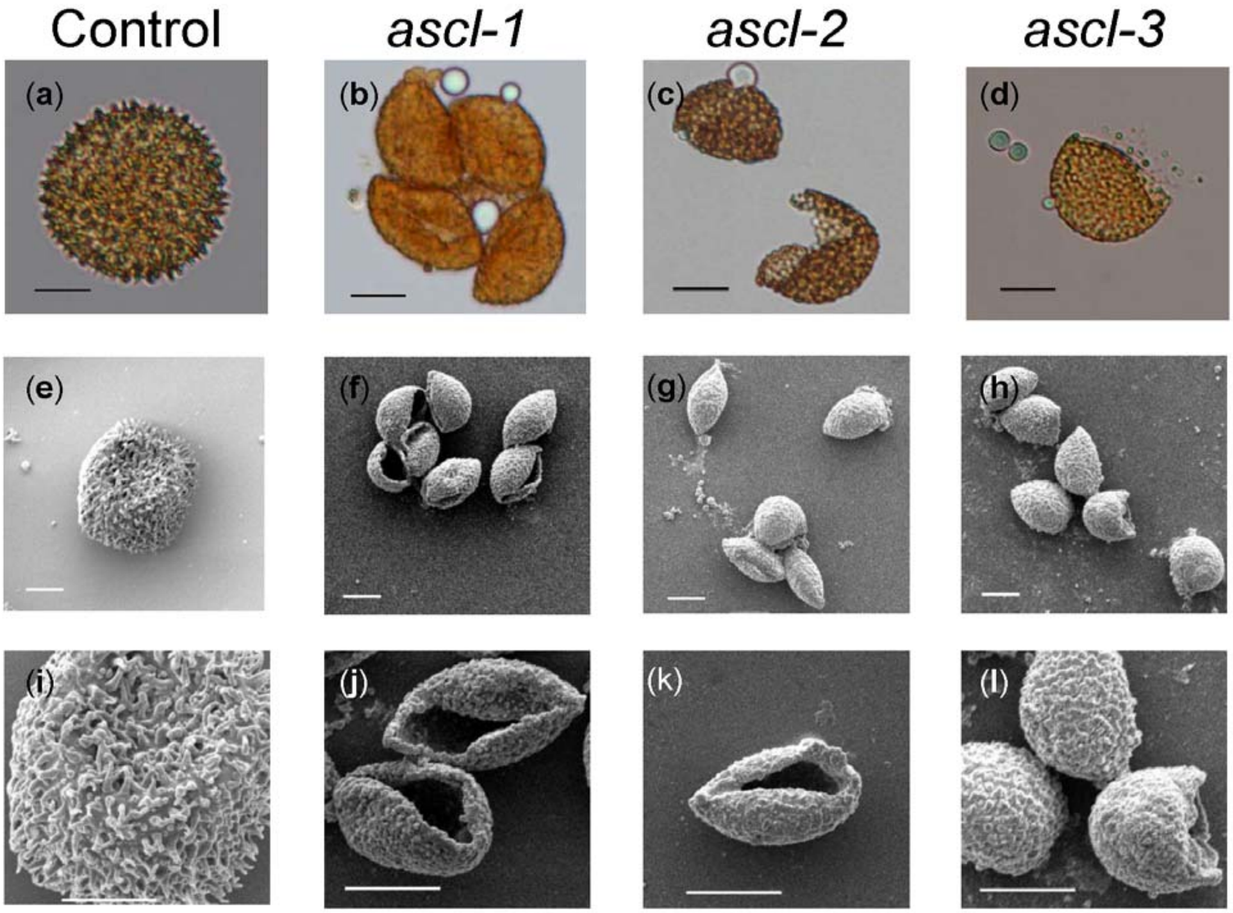

Supplement: S3 Fig — (a–d) Photomicrographs of spores isolated from mature sporophytes. Fractured ascl spore walls are clearly visible in (b) and (c). Ascl plants produced smaller spores. The diameter of control spores was 28 ± 2.0 μm (n = 132, mean ± S.D.); the longest dimension of ascl spores was 20 ± 1.7 μm (n = 63), 18 ± 1.9 (n = 146) and 19 ± 2.2 (n = 39) in ascl-1, ascl-2 and ascl-3, respectively. The ascl spores were significantly smaller than the control spores (ANOVA, p < 0.0001). (e–l) SEM images of fixed, gold-coated spores. All scale bars = 10 μm. (PDF) [file pone.0146817.s003.pdf]
