# Supplementary material for: PpASCL, the Physcomitrella patens Anther-Specific Chalcone Synthase-Like Enzyme Implicated in Sporopollenin Biosynthesis, Is Needed for Integrity of the Moss Spore Wall and Spore Viability
Source: PLoS One. 2016 Jan 11;11(1):e0146817. doi: 10.1371/journal.pone.0146817 (PMC4709238; doi:10.1371/journal.pone.0146817)

S4 Fig. TEM images of control (*pabB4*) and *ascl-2* spores.

Cross sections of control (a) and *ascl-2* (b,c) spores from orange sporophytes were examined by TEM. In the control, the spore wall is intact and the spore is filled with cytoplasm, which here shows little structural resolution except for the faint outlines of oil droplets. The integrity of the spore wall in *ascl-2* has been compromised and the arrows in (b) and (c) indicate the position of an opening or fissure in the wall. The *ascl-2* spores are only partially filled with cytoplasm, presumably because of leakage of cytoplasm from the spores via the fissure in the mutant spore wall. There also appears to be uneven deposition of perine or perine-like material at the surface of the remaining cytoplasm within the *ascl-2* spores. The perine projections on the outside of *ascl-2* spores are smoother and less pointed than those on the outside of control spores. Magnified images of areas outlined in red in (a) and (c) are shown in Fig 7a and b, respectively. Scale bars = 2 μm.

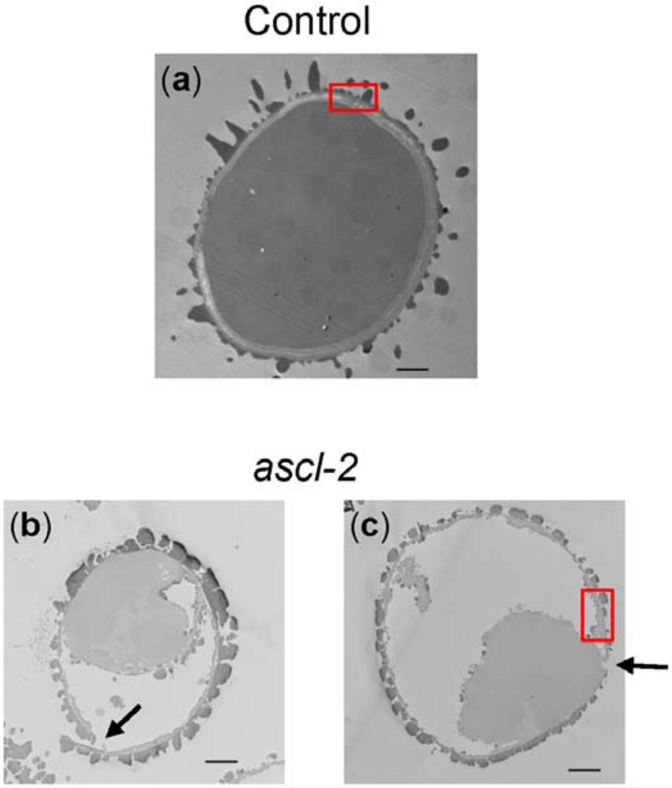

Supplement: S4 Fig — Cross sections of control (a) and ascl-2 (b,c) spores from orange sporophytes were examined by TEM. In the control, the spore wall is intact and the spore is filled with cytoplasm, which here shows little structural resolution except for the faint outlines of oil droplets. The integrity of the spore wall in ascl-2 has been compromised and the arrows in (b) and (c) indicate the position of an opening or fissure in the wall. The ascl-2 spores are only partially filled with cytoplasm, presumably because of leakage of cytoplasm from the spores via the fissure in the mutant spore wall. There also appears to be uneven deposition of perine or perine-like material at the surface of the remaining cytoplasm within the ascl-2 spores. The perine projections on the outside of ascl-2 spores are smoother and less pointed than those on the outside of control spores. Magnified images of areas outlined in red in (a) and (c) are shown in Fig 7A and 7B, respectively. Scale bars = 2 μm. (PDF) [file pone.0146817.s004.pdf]
