# Supplementary material for: PpASCL, the Physcomitrella patens Anther-Specific Chalcone Synthase-Like Enzyme Implicated in Sporopollenin Biosynthesis, Is Needed for Integrity of the Moss Spore Wall and Spore Viability
Source: PLoS One. 2016 Jan 11;11(1):e0146817. doi: 10.1371/journal.pone.0146817 (PMC4709238; doi:10.1371/journal.pone.0146817)
Supplement: S1 Table — (PDF) [file pone.0146817.s005.pdf]

S1 Table. Primer sequences.

Primer	Sequence (5′ → 3′)	Primer binding site[a]
Linear knockout construct generation[b]		
5′F-ASCL-ClaI	GCACGGATCGATTAATGTTGGAAGTTGTGATTGGAAT	–549...–522[c]
5′R-ASCL-ClaI	GCACGGATCGATGCTCGTCCAAAATTTCTTTGTT	422...446
3′F-ASCL-NdeI	GGAATTCCATATGCTACGGGGGCGCTTCT	694...712
3′R-ASCL-NdeI	GGAATTCCATATGACACCAGGACCGAAGGCAA	1430...1450
PCR analysis of stable transformants		
ASCL-gDNA-F	CTTACACATCACTCACTTATTCTGCTGCTT	–792...–763
ASCL-gDNA-R	GAGAATAATACGATCCCTCTCTCAGTTTGA	1661...1690
pTN182-5′-R	TTGAAAAGTGTCAATAGCCCTTTGG	–380...–356[d]
pTN182-3′-F	ATTGGTATCAGGGCCATGAATAGGT	1416...1440
Southern blot analysis		
pTN-G418-F	GTGGAGAGGCTATTCGGCTATGACT	46...70
pTN-G418-R	AACTCGTCAAGAAGGCGATAGAAGG	764...788
RT-PCR		
ASCL-RT-F	AACGACCATGGCAAGTCGAAGGGTCGAGGCG	–7...24
ASCL-RT-R	ACGTGCTGCTGCTGATGTTCCCG	1314...1336
Actin3-F	ATGGCTGGAGAGGGTGAGGATGTCC	1...25[e]
Actin3-R	CCACATCTGCTGGAACGTACTCAGCG	1236...1261

[a] Nucleotide numbering is in relation to the translation initiation codon, starting with number 1 at the A of the ATG.

[b] *PpASCL* locus-specific regions are underlined and restriction sites are highlighted.

[c] *PpASCL* gene sequence (locus name, Phpat.002G137300) is available in the Phytozome database (phytozome.jgi.doe.gov/).

[d] Gene accession number of pTN182 is AB267706. Nucleotide numbering of pTN182 based primers is in relation to the translation initiation codon of *nptII*.

[e] Gene accession number of *Physcomitrella Actin3* is AY382283.
