# Supplementary material for: PpASCL, the Physcomitrella patens Anther-Specific Chalcone Synthase-Like Enzyme Implicated in Sporopollenin Biosynthesis, Is Needed for Integrity of the Moss Spore Wall and Spore Viability
Source: PLoS One. 2016 Jan 11;11(1):e0146817. doi: 10.1371/journal.pone.0146817 (PMC4709238; doi:10.1371/journal.pone.0146817)
Supplement: S2 Table — (PDF) [file pone.0146817.s006.pdf]

S2 Table. Descriptions of developmental stages of sporophytes of *pabB4*, the untransformed control strain.

Individual developmental stages of *pabB4* sporophytes are illustrated in Fig. 4A–N. Additional data about the timing of these developmental stages are provided in **S3 Table**.

(1) *Initial development stage* (Fig. 4A)
A zygote, and subsequent embryo derived from it, grow and develop within the confines of an archegonium, causing the archegonium to expand. In *pabB4*, although there are usually several archegonia containing a fertilized egg at the apex of each gametophore, typically only a single zygote develops into a sporophyte on each gametophore.
(2) *Expanding capsule stage* (Fig. 4B)
It begins at approximately day 10 (after irrigation) with formation of a reddish-brown seta, which connects the capsule to a sporophytic foot embedded in the apex of a gametophore. As the capsule of each embryonic sporophyte develops, its expansion ruptures the archegonial wall transversely. The apical portions of the ruptured archegonium become a calyptra, which sits loosely on the apex of the expanding capsule. The calyptra is easily dislodged but one is visible in Fig. E. As expansion continues, the capsule becomes more spherical and an air space is formed between the capsule wall and the central columella and spore-sac. The majority of sporophytes complete this stage by day 15.
(3) *Green stage* (Fig. 4C,E,G)
Spores become visible within the sporophytic capsule around day 15 and this marks the beginning of the green stage which lasts approximately until day 20. In backlit capsules, developing spores can be seen as a slightly opaque mass through the relatively transparent, green capsule wall. As the spores continue maturing, they become more opaque and, by the end of this stage, the white mass of spores appears to occupy most of the space inside the capsule. Spore mother cells, comprising a layer surrounding the sterile columella, undergo meiosis to form tetrads of meiospores, which are released as free microspores by the end of this stage.
(4) *Yellow stage* (Fig. 4I)
Following the green stage, a relatively short (approximately day 21 to 24) yellow stage occurs, as spores begin to acquire pigmentation. Spores from the yellow stage are larger than those present at the green stage and initial cell wall patterning is apparent.
(5) *Orange stage* (Fig. 4K,M)
It begins at about day 25. This was the last stage observed in *pabB4* sporophytes and persisted for around 48 days, after which the capsular wall ruptured. Spores reach their maximum size during the early orange stage, while pigmentation continues to darken throughout this stage. Dehiscence releasing the mature spores results from tearing of the capsule wall, perpendicular to its longitudinal axis.
