# Supplementary material for: PpASCL, the Physcomitrella patens Anther-Specific Chalcone Synthase-Like Enzyme Implicated in Sporopollenin Biosynthesis, Is Needed for Integrity of the Moss Spore Wall and Spore Viability
Source: PLoS One. 2016 Jan 11;11(1):e0146817. doi: 10.1371/journal.pone.0146817 (PMC4709238; doi:10.1371/journal.pone.0146817)
Supplement: S3 Table — The number of sporophytes at each developmental stage is given as a percentage of the total number in a culture tube. (PDF) [file pone.0146817.s007.pdf]

S3 Table. Raw data of sporophytic development.

The number of sporophytes at each developmental stage is given as a percentage of the total number in a culture tube. Percentage values are presented in a green-yellow heat map where the shade of green indicates higher values and that of yellow lower values.

(1) *ascl-2*

ascl-2		Percentage of sporophytes at each stage						
Days after irrigation	Total SP	Initial develop-ment	Capsule elongation	Green	Yellow	Orange	Dehisced	Brown
6	53	96	0.0	1.9	0.0	1.9	0.0	0.0
7	31	94	0.0	0.0	3.2	0.0	0.0	3.2
10	17	59	41	0.0	0.0	0.0	0.0	0.0
12	24	21	79	0.0	0.0	0.0	0.0	0.0
12	6	50	50	0.0	0.0	0.0	0.0	0.0
12	45	44	44	2.2	4.4	4.4	0.0	0.0
14	65	12	68	20	0.0	0.0	0.0	0.0
15	37	46	35	19	0.0	0.0	0.0	0.0
17	85	5.9	19	69	0.0	3.5	2.4	0.0
17	32	19	25	56	0.0	0.0	0.0	0.0
18	15	20	20	60	0.0	0.0	0.0	0.0
19	76	1.3	5.3	90	1.3	2.6	0.0	0.0
20	58	6.9	22	57	14	0.0	0.0	0.0
21	20	10	45	10	35	0.0	0.0	0.0
21	57	1.8	12	46	26	3.5	3.5	7.0
22	19	16	11	32	42	0.0	0.0	0.0
22	56	3.6	8.9	43	41	3.6	0.0	0.0
25	66	1.5	3.0	7.6	15	73	0.0	0.0
25	58	3.4	10	22	17	41	0.0	5.2
26	46	8.7	17	11	17	46	0.0	0.0
27	54	3.7	3.7	24	5.6	56	0.0	7.4
30	58	3.4	0.0	3.4	8.6	76	0.0	8.6
33	37	30	2.7	8.1	5.4	54	0.0	0.0
37	70	4.3	2.9	1.4	0.0	87	1.4	2.9
48	57	7.0	7.0	5.3	1.8	47	0.0	32
52	47	0.0	0.0	0.0	0.0	79	0.0	21
52	41	2.4	4.9	17	7.3	39	2.4	27
59	89	10	6.7	3.4	1.1	53	2.2	24
60	63	13	0.0	0.0	0.0	59	6.3	22
65	54	5.6	7.4	1.9	1.9	67	1.9	15
68	47	19	0.0	2.1	0.0	55	6.4	17
68	25	0.0	0.0	4.0	0.0	52	4.0	40

(2) Control (*pabB4*)

Control		Percentage of sporophytes at each stage						
Days after irrigation	Total SP	Initial develop-ment	Capsule elongation	Green	Yellow	Orange	Dehisced	Brown
6	53	96	1.9	0.0	1.9	0.0	0.0	-
7	53	77	1.9	1.9	3.8	11	3.8	-
10	18	56	44	0.0	0.0	0.0	0.0	-
12	63	37	57	0.0	0.0	6.3	0.0	-
12	22	23	73	4.5	0.0	0.0	0.0	-
13	61	53	39	4.9	0.0	0.0	3.3	-
14	57	14	74	12	0.0	0.0	0.0	-
15	13	31	23	46	0.0	0.0	0.0	-
15	68	15	43	40	1.5	1.5	0.0	-
17	82	7.3	20	71	0.0	2.4	0.0	-
18	16	13	31	56	0.0	0.0	0.0	-
18	18	11	17	72	0.0	0.0	0.0	-
19	52	13	15	66	0.0	3.8	0.0	-
19	77	3.9	9.1	83	0.0	3.9	0.0	-
20	29	6.9	28	66	0.0	0.0	0.0	-
21	47	2.1	13	34	51	0.0	0.0	-
21	22	0.0	23	55	23	0.0	0.0	-
21	91	4.4	9.9	37	26	8.8	12	1.0
22	23	13	17	30	39	0.0	0.0	-
22	46	4.3	17	37	37	4.3	0.0	-
24	31	13	6.5	26	55	0.0	0.0	-
25	77	3.9	2.6	20	38	36	0.0	-
25	91	3.3	5.5	9.9	37	41	3.3	-
27	65	1.5	4.6	3.1	22	69	0.0	-
27	84	4.8	1.2	9.5	16	69	0.0	-
30	71	2.8	5.6	4.2	7.0	80	0.0	-
31	88	6.8	0.0	10	13	71	0.0	-
31	34	5.9	5.9	0.0	15	74	0.0	-
33	48	29	6.3	10	4.2	48	2.1	-
39	87	14	5.7	4.6	8.0	68	0.0	-
44	56	18	5.4	1.8	3.6	70	1.8	-
46	94	4.3	1.1	2.1	4.3	81	7.4	-
48	64	6.3	11	22	14	47	0.0	-
52	82	11	4.9	3.7	6.1	34	40	-
52	37	0.0	0.0	2.7	8.1	32	57	-
53	29	0.0	10	10	24	35	21	-
65	47	0.0	0.0	0.0	0.0	13	87	-
95	31	9.7	3.2	3.2	3.2	32	48	-
